# Lost in Transition: A Case Study of Oral Baclofen Withdrawal Presenting as Severe Pain

**DOI:** 10.7759/cureus.112805

**Published:** 2026-07-16

**Authors:** Kelli LaCroix, Purvee Patel, Omar Selod

**Affiliations:** 1 Physical Medicine and Rehabilitation, Baylor Scott and White All Saints Medical Center, Fort Worth, USA; 2 Osteopathic Medicine, Texas College of Osteopathic Medicine, University of North Texas Health Science Center, Fort Worth, USA; 3 Physical Medicine and Rehabilitation, Baylor Scott and White Health, Fort Worth, USA

**Keywords:** baclofen withdrawal, hyperalgesia-like pain, medication reconciliation, multi-modality pain management, oral baclofen, spinal cord injury (sci)

## Abstract

Baclofen, a gamma-aminobutyric acid type B (GABA-B) receptor agonist commonly used for spasticity management, is known to cause withdrawal symptoms after abrupt discontinuation. Unique clinical presentations of oral baclofen withdrawal are not well documented, and severe hyperalgesia-like pain disproportionate to expected postoperative recovery has not been well described in the context of oral baclofen withdrawal. This case study depicts a rare presentation of oral baclofen withdrawal in a 46-year-old woman with complete paraplegia and chronic spasticity (Modified Ashworth Scale Grade 4) following an inadvertent 75% dose reduction in her home baclofen regimen (postoperative day 0) at an outside facility during transitions between healthcare facilities.

The patient presented with agitation, tachycardia, diaphoresis, insomnia, severe postoperative lower extremity pain, increased pain sensitivity out of proportion to expected postoperative recovery, and progressive muscle spasms after ankle surgery one week prior. On Hospital Day 1, she required 141.25 oral morphine equivalents (OMEs), including scheduled, one-time, and as-needed opioid doses. Her pain symptoms were worsening despite escalating opioid therapy and the absence of postoperative signs of infection or hardware malfunction on Hospital Day 2. A detailed medication reconciliation on Hospital Day 3 revealed that her chronic baclofen dose of 20 mg four times daily was mistakenly reduced to 20 mg once daily on the day of her surgery. She was given two additional doses of baclofen 20 mg on Hospital Day 3, and her OME was 117.5. Following restoration of her full home baclofen regimen on Hospital Day 4, the patient experienced significant improvement in pain, spasticity, sleep quality, and a proportionate decrease of opioid requirements (OME of 75).

This case highlights the diagnostic challenge of oral baclofen withdrawal when it presents as refractory postoperative pain and emphasizes the clinical finding of hyperalgesia-like pain and rebound spasticity as a potentially unrecognized presentation of oral baclofen withdrawal. Further, it highlights the importance of accurate medication reconciliation, continuity of antispasmodic therapy, and patient education during transitions of care to prevent avoidable complications of baclofen withdrawal and unnecessary escalations of analgesic management.

## Introduction

Baclofen is a gamma-aminobutyric acid type B (GABA-B) receptor agonist commonly used in the management of spasticity associated with conditions such as spinal cord injury, multiple sclerosis, and cerebral palsy. Baclofen may be administered orally or intrathecally, with oral formulations being more widely prescribed due to the ease of administration and lower procedural risk. Oral baclofen often requires multiple daily doses in chronic therapy, and abrupt discontinuation can precipitate a withdrawal syndrome due to neuronal hyperexcitability following the loss of tonic inhibitory control attributed to GABA-B agonism. Symptoms typically emerge within 24-72 hours and can last up to two weeks, depending on maintenance dose, duration of therapy, and timing of baclofen reinitiation [1]. Adult manifestations of oral baclofen withdrawal commonly include rebound spasticity, muscle rigidity, myalgias, agitation, delirium, hallucinations, insomnia, autonomic instability, hyperthermia, and seizures [2].

Intrathecal baclofen withdrawal has been more extensively characterized in the literature due to early manifestations (can be within a few hours) of severe symptoms such as altered mental status, autonomic instability, and multiorgan failure [1-2]. Although the presentation is similar to that of intrathecal baclofen, oral baclofen withdrawal remains less frequently reported and may therefore be underrecognized in clinical practice. Published case reports describe presentations such as hyperactive delirium [3], respiratory failure [4-5], and severe bradyarrhythmia with reduced cardiac output following abrupt cessation of oral baclofen therapy [6], but hyperalgesia-like pain manifestations of oral baclofen withdrawal remain poorly characterized, with severe spasticity-related pain only described with intrathecal withdrawal [7-8]. There are currently no published case reports describing hyperalgesia-like pain presentations in the setting of baclofen withdrawal. This report describes an atypical presentation of oral baclofen withdrawal in a patient with complete paraplegia (T5 American Spinal Injury Association (AISA) Impairment Score A) who had been maintained on baclofen therapy for approximately two years (baseline Modified Ashworth Scale Grade 4). In post-acute care settings, increased pain, agitation, insomnia, and increased spasticity can be easily attributed to etiologies other than medication withdrawal. Recognition of similar simultaneous presentations is important, as delayed diagnosis may prolong patient suffering and lead to unnecessary escalation of pain management in patients with underlying chronic spasticity.

## Case presentation

This is a 46-year-old female who presented to the emergency department from a skilled nursing facility, in which she was placed following a left ankle open reduction internal fixation at an outside hospital approximately one week before presentation. Her main complaints were nausea, vomiting, and lower abdominal discomfort for one to two days, as well as severe left lower extremity pain that had significantly worsened over the last three to four days despite opioid analgesics in addition to her baseline home pain management regimen.

She had a pertinent past medical history of complete paraplegia (AISA Impairment Scale: T5 AIS A) due to a methicillin-sensitive *Staphylococcus aureus* (MSSA) thoracic epidural abscess status-post T4-T10 decompressive laminectomies two years prior, chronic spasticity (baseline Modified Ashworth Scale Grade 4), neurogenic bladder with a suprapubic catheter in place, recurrent urinary tract infections with extended-spectrum beta-lactamase (ESBL) *E. coli* and *Klebsiella*, urosepsis with recent hospitalization just prior to her most recent admission for left ankle fracture, chronic constipation, chronic back pain, insomnia, and bipolar disorder. Her medication list at admission included baclofen 20 mg daily, gabapentin 600 mg three times daily, duloxetine 30 mg twice daily, oxybutynin 5 mg daily, tamsulosin 0.4 mg nightly, lubiprostone 8 mcg twice daily, oxycodone 10 mg every four hours as needed for postoperative pain, divalproex 500 mg nightly, and trazodone 100 mg nightly.

In the ED, she was tachycardic and diaphoretic, but her vital signs were stable. She did not meet systemic inflammatory response syndrome (SIRS) criteria and was afebrile without leukocytosis. Her urinalysis (UA) showed >50 WBCs (Reference range: 0-5/hpf), 4+ bacteria (Reference range: None), 3+ leukocyte esterase (Reference range: Negative), mucus (Reference range: Absent), and 6-10 squamous cells (Reference range: 0-2/hpf), consistent with prior UAs in the context of her suprapubic catheter. The remainder of her workup in the ED was unremarkable. Upon hospital admission (Hospital Day 1), she was very concerned about her left lower extremity pain after her surgery and insisted that her wound be checked. She denied chest pain and shortness of breath. On exam, she was diaphoretic, tachycardic, and wheelchair-bound with bilateral lower extremity strength diffusely grade 0-1 by manual muscle testing. At baseline, she had no bilateral lower extremity sensation, but on exam, she reported feeling a vague pain sensation with palpation of her left lower extremity. Her left ankle surgical wound was clean, dry, and intact without any obvious signs of infection. She was anxious with pressured speech and emotional lability when trying to describe her pain and symptoms. Her electrocardiogram (EKG) and chest X-ray (CXR) were unremarkable. Doppler US of the bilateral lower extremities showed no evidence of deep vein thrombosis, and CT chest showed no evidence of pulmonary embolism. Given her history of ESBL urosepsis and UA suggesting urinary tract infection (UTI), intravenous (IV) meropenem was initiated for empiric coverage. For pain management, she was given her home pain regimen of gabapentin and duloxetine with additional scheduled oxycodone and as-needed methocarbamol and IV Dilaudid® for severe breakthrough pain. The patient frequently requested pain medications and would become very irritable if additional pain medications were not readily available. She was given additional gabapentin 300 mg in addition to her baseline neuropathy dose of 600 mg three times daily that did not provide any relief. Unfortunately, attempts to obtain collateral information were unsuccessful, and her medication reconciliation and history were largely obtained through review of available electronic medical records due to significant patient agitation. 

On Hospital Day 2, the patient remained agitated and reported her insomnia was more severe than usual due to leg pain at night. Her left lower extremity pain was described as severe, generalized, and “difficult to explain” due to the alteration of sensation in the context of her partial paraplegia. Her left lower extremity was exquisitely painful to light palpation and clinically consistent with hyperalgesia-like pain. Repeat X-ray of the left ankle confirmed proper hardware placement with diffuse soft tissue edema attributable to her recent ankle fixation. After discussion with the patient, she was given scheduled methocarbamol and oxycodone and was transitioned to oral Dilaudid for more prolonged pain control, with IV Dilaudid reserved for severe breakthrough pain. In addition to home trazodone 100 mg, she was given ramelteon 8 mg before bedtime for insomnia. She continued to receive IV antibiotics, and her nausea, vomiting, and lower abdominal discomfort resolved.

On Hospital Day 3, she had persistent severe left lower extremity pain and trouble sleeping overnight due to painful bilateral lower extremity spasms. She had visible left lower extremity spasms of the hip, knee, and ankle on exam. The patient was again questioned about her home medication regimen, and a discrepancy arose when reconfirming her home baclofen dose. The patient recalled taking more than 20 mg once daily, the dosage noted in the electronic medical record. A repeat detailed review of her home medication regimen revealed a discrepancy between the documented home dose of baclofen and what the patient recalled taking at home before her hospital admission 11 days prior. With pharmacy assistance, it was confirmed that the patient had filled prescriptions for a home dose of baclofen 20 mg four times daily, leading up to the day of her left ankle fixation. At the time of admission for her ankle surgery one week prior, baclofen 20 mg once daily was listed as her current medication, and it was resumed at this dose following her surgery, effectively serving as an abrupt 75% dose decrease. She was given two additional doses of baclofen 20 mg for a total of three doses, and baclofen was subsequently scheduled as 20 mg four times daily starting the following day, Hospital Day 4.

**Figure 1:**
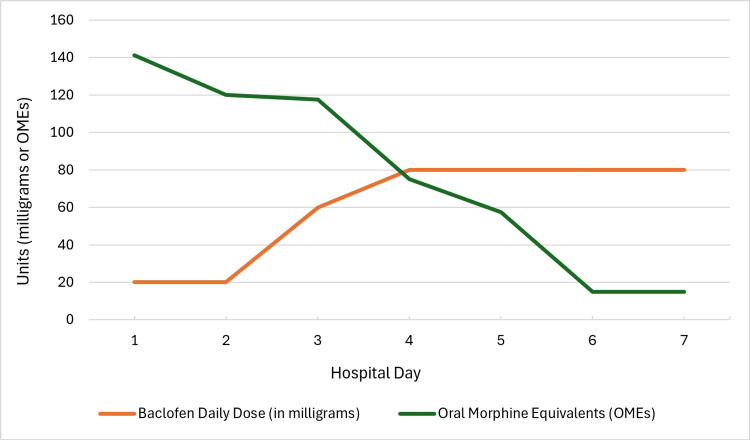
Association between oral baclofen dose and oral morphine equivalents during hospitalization

In the following days, the patient reported that her pain had significantly improved, and she was able to sleep much better at night because her lower extremity spasms were not as frequent or intense. Her pain continued to improve, and she was able to wean down her opioid requirements. For a timeline summary of events, including daily oral morphine equivalents used for pain management, please see Table 1. After the completion of her IV antibiotics course for her UTI, she was discharged on Hospital Day 7 of hospitalization to an inpatient rehabilitation facility. At the time of discharge, she was counseled on the importance of documenting her home spasticity regimen to aid in future medication reconciliations. Figure 1 shows the association between oral baclofen dose and oral morphine equivalents during hospitalization.

**Table 1:** Summary of patient timeline OME = Oral morphine equivalents (including scheduled doses, one-time doses, and as-needed doses). Conversion factors used: Hydrocodone factor = 1, Hydromorphone factor = 4, Morphine = 1, Oxycodone factor = 1.5.

Day	Summary	Opioid Requirements (OMEs)
Preoperative Baseline	Baclofen 20 mg four times a day (80 mg daily); Chronic stable spasticity management	0
Postoperative Day 0	Admission to outside hospital for left ankle surgery; Baclofen documented as 20 mg daily; Effective 75% reduction from home dose resumed after surgery is complete	Unknown
Postoperative Days 1-3	Discharge from outside hospital to skilled nursing facility; Emergence of tachycardia, agitation, and insomnia; Onset of severe left lower extremity pain	Unknown
Postoperative Days 4-10	Persistent severe pain despite escalating opioid therapy; Transfer from skilled nursing facility to hospital	Unknown
Hospital Day 1	Admission to hospital; Ongoing severe pain and sleep disturbance; Development of painful left lower extremity spasms	141.25
Hospital Day 2	Ongoing severe pain, sleep disturbance, and worsening left lower extremity spasticity; X-ray of the left ankle showing hardware in place, no signs of postoperative infection	120
Hospital Day 3	Ongoing severe pain, sleep disturbance, and bilateral lower extremity spasticity; Medication reconciliation discrepancy identified; Additional 20mg baclofen doses administered (60 mg daily total)	117.5
Hospital Day 4	Home baclofen regimen fully restored (80 mg daily total); Sleep quality is improved	75
Hospital Days 5-7	Continued improvement in pain and sleep; Decreased frequency and severity of spasms; Reduced opioid requirements; Discharge to inpatient rehabilitation on Hospital Day 7	Day 5: 57.5; Day 6: 15; Day 7: 15

## Discussion

The most predominant symptoms of baclofen withdrawal exhibited by this patient were initially tachycardia, agitation, and insomnia, followed by progressive hyperalgesia-like pain and rebound spasticity. These symptoms presented in the days following an abrupt 75% decrease in her home baclofen dose from 80 mg daily (maximum recommended daily dose) to 20 mg daily, which the FDA particularly warns against [9].

Based on the patient’s history and physical exam findings, it appears her withdrawal symptoms began to emerge three days after her dose reduction and continued to worsen beyond the first week. While her prolonged symptoms of withdrawal could have been due to long-term use of the maximum oral dose recommended in spasticity management [9], it is interesting to note that the patient was concurrently taking divalproex for bipolar disorder. Divalproex is an anticonvulsant drug with a GABAergic mechanism of action by increasing GABA synthesis and decreasing GABA breakdown [10]. While non-benzodiazepine anticonvulsants are less commonly reported agents used in baclofen withdrawal [2], it is unclear whether this patient's long-term divalproex therapy influenced the severity or timing of her withdrawal symptoms. Given divalproex's GABAergic properties, it is plausible that concomitant therapy may have modified her clinical presentation; however, evidence supporting such an effect in baclofen withdrawal is limited. As such, this hypothesis is currently speculative and warrants further investigation. Although she experienced gastrointestinal symptoms of nausea, vomiting, and lower abdominal pain that can also be seen with baclofen withdrawal, these symptoms resolved early on with IV antibiotics in the context of her UTI. 

What is of interest in this case is the unanticipated pain management issues in relation to the delay in identifying her oral baclofen withdrawal. While the patient was found to have a UTI, her symptoms were worsening despite three days of broad-spectrum IV antibiotics with meropenem (urine sensitivity confirmed susceptibility), which made UTI-related discomfort less likely. She also showed no clinical signs of surgical site infection on exam and again was on broad-spectrum antibiotics that would have been expected to address a surgical site infection (X-ray of the ankle showed no evidence of hardware complications). The patient explicitly stated her baseline neuropathic pain was distinct from her worsening pain during this hospitalization and escalation of her home gabapentin did not help alleviate her symptoms on Hospital Day 1 or 2. This patient’s recent orthopedic surgery and chronic central pain sensitization related to spinal cord injury are additionally considered factors that were likely contributors to her complicated postoperative pain management course. While hyperalgesia is not specifically mentioned as a symptom of baclofen withdrawal by the FDA or in the recent systematic review by Iqbal et al. [2], potential explanations for this patient's heightened pain sensitivity are rebound spasticity, painful paresthesia, and reduced GABA-B-mediated nociceptive inhibition [11]. Although pain related to worsening spasticity has been reported during baclofen withdrawal, the severe pain sensitivity observed in this patient appeared disproportionate to the expected postoperative course and may represent a less commonly recognized presentation of oral baclofen withdrawal. Exacerbated sleep deprivation due to insomnia and psychological stress in the form of agitation are also pain-exacerbating factors witnessed in this patient that are thought to be attributable to baclofen withdrawal based on the timeline of symptom presentation beginning on postoperative Days 1-3. Despite the escalation of opioid analgesics and multimodal pain management on Hospital Day 1, her pain remained severe and was accompanied by tachycardia, agitation, insomnia, and progressive muscle spasms, which are all recognized features of baclofen withdrawal. Her hyperalgesia-like pain, sleep disturbance, muscle spasms, and opioid requirements decreased abruptly following restoration of her home baclofen dose, supporting baclofen withdrawal as a major contributor to her pain presentation. While muscle pain is noted as a rare adverse reaction to baclofen by the FDA [9], its relationship to baclofen withdrawal remains unclear. Given the biological plausibility of this mechanism, additional clinical investigation is needed to better understand the contribution of baclofen withdrawal to pain sensitization. 

Limitations of this case report include its single-patient design, which limits generalizability to larger patient populations. Multiple potential confounders were present, including recent orthopedic surgery, spinal cord injury, urinary tract infection, and concurrent medications that may have influenced presentation of symptoms and severity of pain. In addition, the proposed association between baclofen withdrawal and out-of-proportion hyperalgesia-like pain is based on clinical observations, and there is no current objective measure of hyperalgesia for this patient that is available. Although the patient's symptoms improved following restoration of her home baclofen dose, suggesting a temporal relationship, a definitive causal relationship cannot be established. The documentation of other objective measures such as hourly pain scales or laboratory values such as creatinine kinase is unfortunately unavailable in this case, but would help strengthen the evidence for baclofen withdrawal among other differential diagnoses. Further studies that include such objective measures are needed to better characterize pain manifestations associated with baclofen withdrawal.

Regardless of the specific pain mechanism, this patient’s withdrawal from baclofen significantly complicated her postsurgical pain management. There was a delay in reinitiating her home baclofen dose after surgery, which highlights the importance of accurate medication reconciliation across patient care settings. This observation is consistent with findings from the MARQUIS2 study, which identified clinician-led medication reconciliation as an effective strategy for reducing medication discrepancies during transitions of care [12]. This highlights a potentially preventable contributor to this patient's prolonged baclofen withdrawal. As mentioned in the case study, this patient had a hospital admission for urosepsis just before her hospital admission for left ankle fracture repair. She was transferred from a skilled nursing facility to an outside hospital for her left ankle fracture, which appears to be the beginning of her baclofen dose discrepancy. She was discharged to a different skilled nursing facility after her surgery before presenting to our hospital, which further complicated her medication reconciliation. Medications are often listed as “no longer taking” in the electronic record when a patient is discharged from a temporary facility, such as a skilled nursing facility. Health care providers must pay close attention to potential medication reconciliation pitfalls, particularly in patient populations requiring both chronic spasticity and pain management. Patients should also be informed of their antispasmodic dosing and the risks of both toxicity and withdrawal from these medications so they can help prevent medication reconciliation errors.

## Conclusions

Rebound spasticity, painful paresthesia, and altered GABA-B-mediated modulation of nociceptive signaling may contribute to increased pain sensitivity and complicate postoperative pain management in spinal cord patients with chronic spasticity. While severe pain and painful spasticity are recognized features of baclofen withdrawal, this case suggests that clinical observation of severe hyperalgesia-like pain out of proportion to the expected postoperative course may represent a less commonly recognized presentation of oral baclofen withdrawal in the presence of additional symptoms such as insomnia and agitation. This presentation is problematic in patients whose pain is difficult to control, and it is important for clinicians to ensure that chronic spasticity medications are appropriately documented during medication reconciliation at the time of each care transition. More research is needed to explore potential pain-related symptoms by including objective pain sensory testing to better characterize the relationship between clinically suspected hyperalgesia-like pain and oral baclofen withdrawal.
